# 

**Published:** 1891-10-10

**Authors:** 


					^SQQtVvW?.S TERfl InTABMARX
mvficti tfosBUAM
WITH EXTRA NURSING SUPPLEMENT.
Saturday, October 10th, 1891

				

